# Five-Year Trend Analysis of Malaria Prevalence in Dembecha Health Center, West Gojjam Zone, Northwest Ethiopia: A Retrospective Study

**DOI:** 10.1155/2020/8828670

**Published:** 2020-11-14

**Authors:** Dessalegne Haile, Aster Ferede, Bekalu Kassie, Abtie Abebaw, Yihenew Million

**Affiliations:** ^1^Department of Nursing, College of Health Sciences, Debre Markos University, Debre Markos, Ethiopia; ^2^Department of Public Health, College of Health Sciences, Debre Markos University, Debre Markos, Ethiopia; ^3^Department of Midwifery, College of Health Sciences, Debre Markos University, Debre Markos, Ethiopia; ^4^Department Medical Laboratory Sciences, College of Health Sciences, Debre Markos University, Debre Markos, Ethiopia; ^5^Department of Medical Microbiology, School of Biomedical and Laboratory Sciences, College of Medicine and Health Sciences, University of Gondar, Gondar, Ethiopia

## Abstract

**Background:**

Malaria is a mosquito-borne infectious disease known to cause significant numbers of morbidities and mortalities across the globe. In Ethiopia, its transmission is generally seasonal and highly unstable due to variations in topography and rainfall patterns. Studying the trends in malaria in different setups is crucial for area-specific evidence-based interventions, informed decisions, and to track the effectiveness of malaria control programs. The trend in malaria infections in the area has not been documented. Hence, this study aimed to assess the five-year trend in microscopically confirmed malaria cases in Dembecha Health Center, West Gojjam Zone, Amhara national regional state, Ethiopia.

**Methods:**

A health facility-based retrospective study was conducted in Dembecha Health Center from February to April 2018. All microscopically confirmed malaria cases registered between 2011/12 and 2015/16 were carefully reviewed from laboratory record books and analyzed accordingly.

**Results:**

A total of 12,766 blood films were requested over the last five years at Dembecha Health Center. The number of microscopically confirmed malaria cases was 2086 (16.34%). The result showed a fluctuating yet declining trend in malaria infections. The highest number of cases was registered in 2012/13, while the lowest was in 2015/16. Males and age groups >20 constituted 58.9% and 44.2% of the patients, respectively, being the hardest hit by malaria in the area. Malaria existed in almost every month and seasons. *Plasmodium falciparum* was the predominant species. The highest peak of malaria infections was observed in the late transition (October-December) 799 (38.3%) and early transition (May-June) 589 (28.2%) seasons.

**Conclusion:**

Although the results indicate a fluctuating yet declining trend, the prevalence of confirmed malaria cases in the area remains alarming and indicates a major public health burden. Therefore, close monitoring and intervention measures to control malaria infections in the area and also to tackle the dominant species, *Plasmodium falciparum*, are necessitated accordingly.

## 1. Background

Malaria is one of the major global public health problems affecting different segments of the population, commonly women and children, and an estimated 228 million cases of malaria occurred worldwide in the year 2018 [1]. The WHO African Region carries a disproportionately high share of the global malaria burden, home to 93% of malaria cases and 94% of malaria deaths [2].

The disease is transmitted by the bite of the infected female *anopheles* mosquito. Infections may result in a wide variety of symptoms ranging from absent or very mild symptoms to severe diseases leading to life-threatening conditions such as cerebral malaria, organ failure, pulmonary oedema, and even death [3]. *Plasmodium falciparum* and *P. vivax* are the most predominant parasites responsible for the disease accounting 60 and 40% of the cases, respectively [4]. The two main malaria transmission seasons in the region are encountered following the small rain season (May-June) and following the major rain season (September-December) [5].

The implementing various malaria control interventions in Ethiopia such as the use of insecticide-treated bed nets, indoor residual spraying, and treatment of cases with arthemicinin-based combination therapies resulted in promising improvements [6, 7]. Despite the achievement, unstable malaria transmission patterns with other contributing factors make an estimated 68% of the population are at risk of contracting malaria [7, 8] and 2,927,266 new malaria cases and 4782 deaths [9].

Dembecha is one of the malaria-prone areas in the region, and according to the Dembecha Health Center report, malaria is among the top ten leading causes of morbidity in the area. Although malaria is the top listed reason for the health center's visit and admissions in the area, there are no documented studies. Further, small and mobile subpopulation groups that are difficult to track may reintroduce malaria in areas where it had been eliminated [10]. Thus, ensuring sufficient data from region of unstable malaria transmission will greatly contribute to the development of strategic control initiatives. Therefore, this study aimed to assess a five-year trend of malaria along seasonal patterns and distributions over sex and age in Dembecha health, northwest Ethiopia.

## 2. Methods and Materials

### 2.1. Study Area

The study was conducted in Dembecha Health Center. Dembecha is a town in northwestern Ethiopia, 350 km north of Addis Ababa. Located in the West Gojjam Zone of the Amhara Region, this town has a latitude and longitude of 10°33′N and 37°29′E with an elevation of 2083 meters above sea level. The district has 19 health centers and 4 private clinics. The Dembecha Health Center was established in 2004 and has an estimated catchment population of 250,000. Malaria is endemic in the area, but it shows seasonal variation. The major malaria transmission periods occur from September to December, following the rainy summer seasons and from March to May.

### 2.2. Study Design

A retrospective cross-sectional study was conducted between February and April 2018 in Dembecha Health Center.

### 2.3. Data Collection

Five years (Sep. 2011/12-Aug. 2015/16) secondary data were collected from the laboratory record logbook. Variables such as date of examination, total clinically treated and confirmed cases in months and years, types of malaria species, and sociodemographic data such as age and sex were collected. Data was collected by experienced medical laboratory technicians. Any incomplete data such as the sociodemographic and malaria diagnosis results which were not properly documented were excluded. In the health center, microscopy was used as the gold standard to confirm the presence of *Plasmodium* parasite by examining peripheral smears of stained blood films (by 10% of Giemsa staining), as outlined in the WHO protocol (WHO, 2010) [11]. The health center follows a standard operating procedure (SOP) for blood smear preparation, staining, and blood film examination for malaria parasite detection.

### 2.4. Data Processing and Analysis

Patients' data including dates of health center visit, sociodemographic characteristics, and laboratory results were entered into EpiData 3.1 and then exported to SPSS version 24 software. Descriptive statistics were used to summarize the data. Line graphs were used to show the trends over the five years. To assure the quality of the data, a well-prepared checklist was used. The training was given for five data collectors and the supervisor. The collected data were checked for completeness and consistency daily. Data cleaning was also done using SPSS. Chi-square test was used to compare the, association of malaria burden by sex and age groups. *P* < 0.05 was considered as statistically significant. The analyzed data was presented using tables and figures.

## 3. Results

### 3.1. Trends in Malaria Cases in Dembecha Health Center

A total of 12,766 blood films were requested over the last five years, 2011/12 and 2015/16 at Dembecha Health Center. The number of microscopically confirmed malaria cases was 2086 (16.34%), and an average of 417 malaria confirmed cases was recorded annually (Table 1).

**Figure 1:**
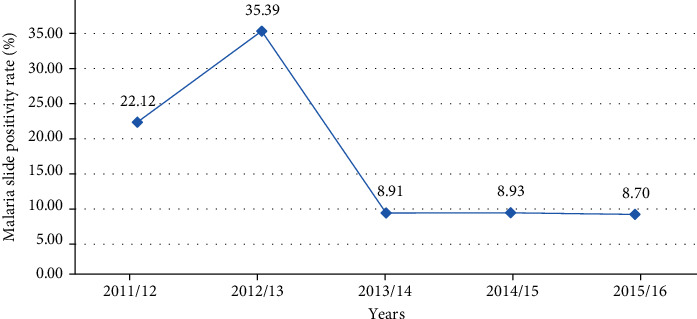
Trends of malaria prevalence in Dembecha Health Center, northwest Ethiopia (2011/12-2015/16).

Results from the record review showed a fluctuating trend in the malaria cases within the past five years. Between the years 2011/12 and 2012/13, a statistically significant increment of malaria morbidities was seen followed by a sharp decline between the years 2012/13 and 2013/14 (*χ*^2^ = 943.91, *d*.*f*. = 4, *P* < 0.01). From 2013/14 to 2014/15, the number of cases remained relatively constant. On the other hand, in the following two years, a decrement in the number of cases was observed. The maximum number of confirmed malaria cases reported was 739 (35.4%) in the year 2012/13. In contrast, the minimum number of confirmed malaria cases registered was 183 (8.7%) in 2015/16 (Figure 1).

### 3.2. Prevalence of Confirmed Malaria Cases in Relation to Sex and Age

Overall, out of 2086 confirmed malaria cases, males [1229 (58.9%)] were more affected by malaria infection than females [857 (41.1%)] in all the years (*χ*^2^ = 4.82, *d*.*f*. = 1, *P* = 0.028). *P. falciparum* infections were the predominant ones in both males and females within these five years (*χ*^2^ = 9.642, *d*.*f*. = 3, *P* = 0.022). Mixed infections were also reported more in males than in females (Table 2).

### 3.3. *Plasmodium* Species Distribution with Age Groups

**Figure 2:**
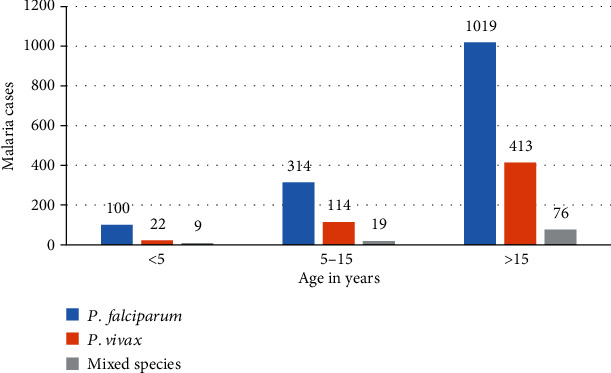
Trends of malaria species across age groups in at Dembecha Health Center, northwest Ethiopia (2011/12-2015/16).

Regarding the trends in malaria species across the age groups, results depicted that in all age groups, *P. falciparum* was the predominant species followed by *P. vivax*. A sharp increment of cases in these two species was observed in the age groups 10-15 through >20 years. An increment in mixed infections was also seen in the age groups 10-15 years through >20 (Figure 2).

### 3.4. *Plasmodium* Species Distribution

**Figure 3:**
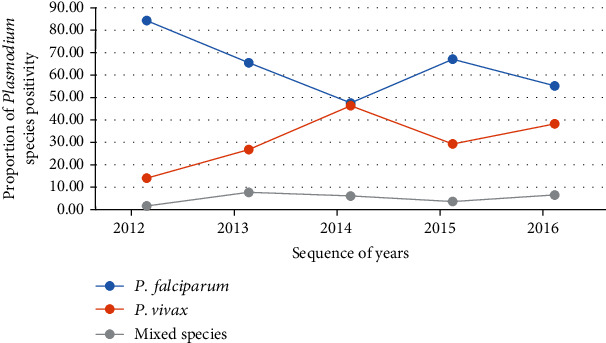
*Trends of malaria species in* at Dembecha Health Center, northwest Ethiopia (2011/12-2015/16).

According to our record review, *Plasmodium falciparum*, *Plasmodium vivax*, and mixed infections (*Plasmodium falciparum* + *Plasmodium vivax*) were reported accounting for 1433 (68.7%), 549 (26.3%), and 104 (5%) of malaria morbidity, respectively (*χ*^2^ = 160.789, *d*.*f*. = 8, *P* < 0.001). The predominant species within the five years was *Plasmodium falciparum.* Morbidities related to this species decreased slightly between 2011/12 and 2012/13, which was followed by a sharp decrement from 2012/13 and 2013/14. In the remaining years, the trend was relatively constant except for the significant rise in 2014/15. Infections with *Plasmodium vivax* and mixed infections (*Plasmodium falciparum* + *Plasmodium vivax*) exhibited increment from 2011/12 to 2012/13, and in the succeeding years, the numbers showed a relative decrement (Figure 3).

**Figure 4:**
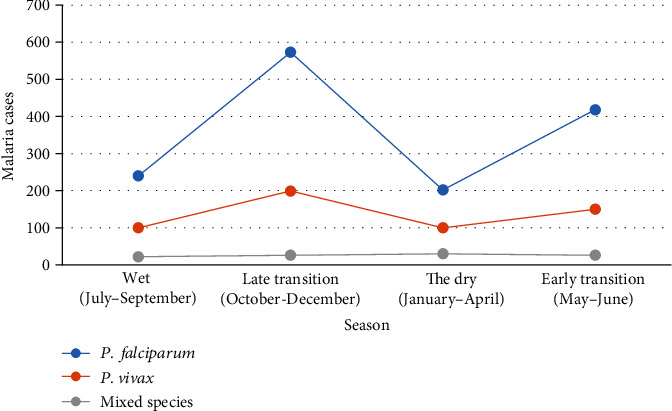
The distribution of *Plasmodium* species in different seasons at Dembecha Health Center from 2011/12 to 2015/16.

Despite the rise and fall seen, malaria cases were reported in almost every months and season of the year. Taking a look at the distribution of these species across the season, the maximum number of cases of *p. falciparum* 573 (39.98%) was registered in late transition (October-December) followed by early transition (May-June) 418 (29.17%), while the least number of cases being in the dry season (January-April). *P. vivax* infections were maximum 199 (36.24%) during late transition season followed by early transition 150 (27.32%). Overall, the maximum number of malaria cases of all species were reported in late transition 798 (38.25%) and early transition 594 (28.47%) seasons (*χ*^2^ = 33.063, *d*.*f*. = 6, *P* < 0.001) (Figure 4).

## 4. Discussion

Malaria is a major public health concern in Ethiopia responsible for substantial amounts of morbidities and mortalities. Its distribution and transmission vary from place to place. This document review was aimed to assess the trend in confirmed malaria cases over five years by a person, composition of *Plasmodium* species and season in Dembecha Health Center. A total of 2086 (16.34%) microscopically confirmed malaria cases were registered from 2011/12 to 2015/16. Comparable positive parasitaemia prevalence was also reported in studies conducted in Arsi Negelle [12] and Kombolcha [13]. The figure could be suggestive of the continued occurrence of high malaria causalities and the burden it is imposing in the study area which may require careful attention from the government and other stakeholders. In contrast, studies done in Woreta [14] and Felegehiwot hospital [15] reported a smaller number of malaria cases. This could be attributable to the differences in the mosquito control measures, population vulnerability, nearby water sources, climate, altitude, and screening techniques and procedures followed. On the other hand, a higher number of malaria cases were encountered in south-central Ethiopia [16]; Wolaita [17]; Kola Diba, north Gondar [18]; and Gorgora and Chuahit, north Gondar [19]. This could be explained by the differences in the health facility setup and catchment population they serve, geographical setup, study area, and the study period. For instance, our study included only Dembecha Health Center unlike the study done in Wolaita, which considered reports from six health centers [17].

The trend analysis result showed that malaria cases were fluctuating over the five years but ultimately showed a decline. This fluctuating yet declining trend was consistent with other studies [20, 21]. An increase in the number of cases was observed since 2011/12 and reached its peak in 2012/13. Numerous components might be contributed for the fluctuation of malaria cases: climatic factors, ecologic and natural variables, host and vector variables, and social and economic factors, change in medical services foundation, mosquito control measures, population vulnerability, nearby water sources, availability of health facilities, and drug resistance [22, 23].

Following the steady increment of cases, a sharp decline was observed from 2012/13 to 2013/14. According to the information obtained from the health center, the reduction of morbidities related to malaria coincides with the increased attention given to malaria prevention and control programs and activities in the community after the preceding alarming results. It also matches with the time Ethiopia was among the countries which achieved a high number of insecticide-treated mosquito net deliveries. Similarly, several efforts were underway to control malaria such as enhancing investments in malaria reduction, delivery of rapid diagnostic tests, wide coverage of arthemicinin-based combination therapies, the use of indoor residual spraying, and different vector control programs as the components of scale-up of malaria interventions that were on the go by the Federal Ministry of health of Ethiopia to fit with the global goal to be achieved by 2020 [2, 24, 25]. The declining trend could also be attributed to the increased role of health extension workers in the diagnosis and treatment of malaria with rapid diagnostic tests and arthemicinin combination therapies, in the distribution and follow-up of long-lasting insecticide nets and indoor residual spraying at the community levels.

In our study, *P. falciparum* was found to be the predominant species accounting for 69% of the cases, followed by *P. vivax*. This is in agreement with the national malaria reports that highlighted the dominance of *P. falciparum* infections (and secondly *P. vivax*) over the other species in Ethiopia [4]. Furthermore, P. *falciparum* was the most prevalent pathogen in many other African countries in charge of significant amounts of morbidities and mortalities across the continent [26]. However, studies done in Jimma [27] and Arsi [12] revealed that *P. vivax* was the prevailing cause of infection. This variation could be due to the relatively highland climatic condition of the study area in which *P. vivax* is a prevalent species in the highlands and might be due to the emergence of drug resistant and the parasite's ability to transmit early in the course of the disease and a relapse from dormant liver stages at varying time intervals after the initial infection [28].

According to studies conducted in different parts of Ethiopia, males were found to be more prone to malaria infection as compared to females [29, 30]. The possible justification given for this finding had been the increased involvements of males in outdoor activities. A similar figure was obtained in the current study. The prevailing species in both males and females across the five years was *P. falciparum*, except in 2013/14, at which time *P. vivax* dominated in males. This trend shift of *Plasmodium* species could be due to the increased attention given for *P. falciparum* and underestimating the burdens of *P. vivax*. The other possible reason for its dominance could be its ability to relapse and ultimately increase the number of cases.

Regarding the age group distributions, increased numbers of malaria morbidities were seen among those >20 years. These groups are more prone to malaria, since they are on their productive age and with family responsibilities exposing them for mobilizations to different areas. Furthermore, the majority of them are from rural areas, where most of them are engaged in agricultural activities that are usually conducted outdoor.

The other important finding in our study was that malaria was observed in almost every months of the year. Also, the relative dominance of all malaria cases was noticed during spring (September, October, and November) and autumn (March, April, and May) seasons, respectively. This is line with the fact that in Ethiopia, malaria transmission peaks biannually from September to December (after the predominant rainy season) and from April to May (following the minimal rainfall season), which coincides with the major harvesting seasons [29]. This is of a big implication, since it might affect the economic welfare of the people and economic development of the country which is mainly based in agriculture.

## 5. Conclusion

Our results indicated that the trend in malaria prevalence was fluctuating yet decreasing in the subsequent years. However, the figure implies that malaria is a major public health problem in the study area affecting the productive segments of the population and its occurrence coincides with the major harvesting seasons. Malaria prevention and control programs should be strengthened taking these implications into account. Also, interventions aimed to combat the infection should give due focus to the predominant species. Further studies involving primary data in the study area are also recommended.

## Figures and Tables

**Table 1:** Annual trends in total malaria cases in Dembecha Health Center, northwestern Ethiopia (2011/12-2015/16).

Year	Blood films examined	Lab confirmed malaria cases
2011/12	3,024	669
2012/13	2,088	739
2013/14	2,762	246
2014/15	2,789	249
2015/16	2,103	183
Total	12,766	2086

**Table 2:** Distribution of confirmed malaria cases by sex at Dembecha Health Center, northwest Ethiopia (2011/12-2015/16).

Sex	Total case examined	Slide positive no. (%)	*P. Falciparum* no. (%)	*P. vivax* no. (%)	Mixed no. (%)
Male	7243	1229 (58.9)	826 (67.20)	345 (28.07)	58 (4.71)
Female	5523	857 (41.1)	607 (70.82)	204 (23.80)	46 (5.36)
Total	12,766	2086	1433(68.7)	549 (26.3)	104 (5)

## Data Availability

All datasets on which the conclusions of the manuscript rely are presented in the manuscript.

## Abbreviations

SD: Standard deviation
SPSS: Statistical package for social sciences
WHO: World Health Organization.
